# Continuous monitoring using thiocyanate ion-selective electrodes

**DOI:** 10.1155/S1463924681000539

**Published:** 1981

**Authors:** Takashi Korenaga

**Affiliations:** 1 Saidaiji Plant Japan Exlan Co Ltd Kanaoka-higashi Okayama-shi 704 Japan; 2 School of Engineering Okayama University Tsushima-naka Okayama-shi 700 Japan


### Continuous monitoring using thiocyanate ion-selective electrodes

**Takashi Korenaga***
*Saidaiji Plant, Japan Exlan Co Ltd, Kanaoka-higashi, Okayama-shi, 704 Japan*

### Introduction

Ion-selective electrodes have been widely used in automating analyses for the potentiometric determination of traces as well as high concentrations of anions and cations. However, the use of electrodes for the routine and continuous monitoring of thiocyanate in 'environmental' water samples has not been fully considered. Recently, the author proposed an automated potentiometric determination of thiocyanate at the ppm level in water using an ion-selective electrode with liquid membrane [1]. In that paper, thiocyanate ion-selective membranes were basically examined for electrodes utilising long-chain quaternary ammonium cations such as tetradecyldimethylbenzylammonium and methyltrioctylammonium ions as the exchange sites in order to automate the procedure for the routine analysis of thiocyanate at the ppm level in water.

However, an important additional requirement for such a procedure is that it should be easy to use, so that it would give precise results from a simply constructed apparatus. Hence, the problem was to devise a system which met all these requirements. Furthermore, it would have to be cheap to manufacture, since there are practical advantages in building on a modular basis with one module dedicated to each analysis. In this paper therefore, the practical problems of automating the system using thiocyanate ion-selective electrodes and also the continuous monitoring of thiocyanate in industrial waste water are described. The feasibility of using thiocyanate ion-selective electrodes with solid and liquid membranes for this purpose are evaluated. Also discussed are methods for overcoming difficulties of electrode maintenance during continuous monitoring. The electrodes compared are a commercial bromide ion-selective electrode with silver bromide solid membrane (Toa Model BR-125, obtained from Toa Electronics Ltd, Japan) and a thiocyanate ion-selective with tetradecyldimethylbenzylammonium thiocyanate liquid membrane using 1,2-dichloroethane described in the previous work [1].

### Materials and methods

### Thiocyanate ion-selective electrode with liquid membrane

The liquid membrane containing an ion-associate formed between tetradecyldimethylbenzylammonium (zephiramine; obtained from Dojin Chemical Laboratories Co Ltd, Japan) and thiocyanate was prepared by extraction with 1,2-dichloroethane (DCE). The thiocyanate ion-selective electrode with liquid membrane was then prepared as follows [1]. 100 ml of a $1 \times 10^{-3}$ M sodium thiocyanate aqueous solution was transferred into a 500 ml separation funnel. 100 ml of $1 \times 10^{-3}$ M zephiramine (chloride) in DCE solution (obtained by directly dissolving 36.9 mg of dried zephiramine chloride [2] into 100 ml of DCE) was added and the resulting solution was shaken for 60 mins in an Iwaki Model KM shaker. The aqueous phase was discarded and the organic phase shaken again with another 100 ml aliquot of the $1 \times 10^{-3}$ M sodium thiocyanate aqueous solution in order to purify it. After phase separation, the organic phase was filtered through a dry filter paper to remove droplets of water. The organic solution was then diluted with DCE to give a $1 \times 10^{-4}$ M solution which was used to make the thiocyanate ion-selective liquid membrane. The electrode was constructed using the barrel of an Orion Model 92-07 nitrate ion-selective electrode, and the $1 \times 10^{-4}$ M zephiramine thiocyanate in DCE solution plus a $1 \times 10^{-2}$ M aqueous sodium thiocyanate solution as organic liquid membrane and internal reference solutions respectively. The liquid is supported by a cellulose membrane filter used as a barrier to keep the liquid membrane solution and aqueous sample solution separate. The liquid membrane potentials with a Yokogawa Model MR-Y511 reference electrode are measured using a Hitachi-Horiba Model F-5 pH meter equipped with a Yokogawa Model 3046 laboratory recorder.

*Present address: School of Engineering, Okayama University, Tsushima-naka, Okayama-shi, 700, Japan*

### Procedure for continuous monitoring

A schematic diagram of the continuous thiocyanate monitoring equipment used in this work is given in Figure 1. The waste water sample was pumped up to a filtration unit (C) with an Iwaki Model LP-15 laboratory pump (Figure 1 A) at a flow rate of 5 l/min, which was adjusted by a flow meter (B). The sample was pre-treated by the filtration unit which has three different meshed plastic filters (Figure 1, a: 10 mesh, b: 40 mesh, c: 200 mesh) to avoid contamination of the electrode by suspended solids. The resulting sample solution was then thermostated at 25 ± 0.5°C with a Mitamura circulator-type thermostated unit (D - volume capacity: 5 l) and well stirred by a circulator (circulation capacity: 5 l/min). The sample obtained by these pre-treatments was used for the measurement of thiocyanate under a Denkikagakukeiki ultrasonic wave cleaner (E). The membrane potential, based on the thiocyanate concentration, was detected by a Hitachi-Horiba Model F-5 pH meter (H) equipped with a thiocyanate ion-selective electrode (F) and a Yokogawa Model MR-Y511 reference electrode (G). The data obtained was continuously recorded by a fitted Yokogawa Model 3046 laboratory recorder (I).

### Results and discussion

### Preliminary tests by batch work

Before use in the continuous monitoring system the basic characteristics and performance of the thiocyanate ion-selective electrode was evaluated with both liquid and solid membranes. The electromotive forces of a solid membrane electrode (commercially available bromide ion-selective electrode: Toa Model BR-125) and a liquid membrane electrode obtained according to the previous paper [1], were measured using a pH meter after the constant membrane potentials were obtained. The temperature of sample solutions was controlled at 25±0.5°C, and the solutions were stirred during the measurements.

The response ranges of both electrodes were first examined and it was found that the membrane potentials of both electrodes against the logarithmic activity of the thiocyanate ion held a Nernstian relationship down to $10^{-5}$ M sodium thiocyanate solution (0.58 ppm as $SCN^-$). However, the response range of the liquid membrane electrode was slightly wider than that of the solid one because linearity was maintained at higher concentrations.

The response rate was also examined to determine the time necessary for the membrane potentials to equilibrate. Constant responses were found at 40 and 80 sec respectively when the liquid and solid membrane electrodes were used with $10^{-4}$ M sodium thiocyanate sample solution. In this sense, the thiocyanate ion-selective electrode with liquid membrane was superior to the solid membrane.

**Table 1. Comparison of the selectivity coefficient ($K_j$) of various anions for liquid and solid membrane electrodes**

| Anion | $K_j$* | |
|---|---|---|
| | Liquid membrane electrode | Solid membrane electrode |
| $ClO_4^-$ | 4.0 | $<10^{-4}$ |
| $I^-$ | 1.6 | 50 |
| $SCN^-$ | 1.0 | 1.0 |
| $NO_3^-$ | 0.033 | $<10^{-4}$ |
| $Br^-$ | 0.008 | 10 |
| $ClO_3^-$ | 0.002 | $<10^{-4}$ |
| $Cl^-$ | 0.0001 | 0.02 |
| $HSO_3^-$ | $<10^{-4}$ | 0.03 |
| $CH_3COO^-$ | $<10^{-4}$ | $<10^{-4}$ |
| $HCO_3^-$ | $<10^{-4}$ | $<10^{-4}$ |
| $H_2PO_4^-$ | $<10^{-4}$ | $<10^{-4}$ |
| $SO_4^{2-}$ | $<10^{-4}$ | $<10^{-4}$ |

*The $K_j$ value was estimated as follows. Experiments were carried out to determine the response of other ions (selectivity coefficient) by comparing with the membrane potentials of $10^{-4}$M sodium thiocyanate aqueous solution in the presence and absence of the other ion (ie. j-ion). The response of thiocynate ion-selective electrode in the presence of an interferring j-ion is given by the following equation [5]:

$$E = \text{constant} - 2.303\,(RT/F)\left\{\log a_{SCN} + K_j\,(a_j)^{1/z}\right\}$$

where $a_{SCN}$ and $a_j$ denote the activities of thiocyanate and other interferring j-ions respectively, and where $K_j$ is the selectivity coefficient of j-ion for the thiocyanate ion-selective electrode and z is the charge of the j-ion. The activities of $a_{SCN}$ and $a_j$ in the above equation are given approximately by the molar concentrations since dilute solutions were used.

The effect of pH on the liquid and solid membrane potentials was also examined. The pH was adjusted by using sulphuric acid and sodium hydroxide. The variations of pH had no effect on the membrane potentials over the pH ranges from 1 to 13 and from 2 to 12 against the liquid or the solid membranes. However, both electrodes were found to be affected by a high ionic strength in the sample solutions.

The effect of temperature was examined and both electrodes were found to be affected by variations of sample temperature. The results obtained by varying the sample temperature in the determination of thiocyanate agreed with those obtained by calculation.

The response of both membranes to other ions was studied, and the selectivity coefficients ($K_j$) of other ions were obtained by comparing with the membrane potentials of $10^{-4}$ M sodium thiocyanate aqueous solution in the presence and absence of the other ions of course spiked samples were used. The values of selectivity coefficient obtained for both liquid and solid membrane electrodes are listed in Table 1 confirming their performances for the detection of thiocyanate ion. From $K_j$ values, the order of selectivity for thiocyanate ion using the liquid membrane electrode was $ClO_4^-$ >$I^-$ >$SCN^-$ >$NO_3^-$ >$Br^-$ >$ClO_3^-$ >$Cl^-$ >$HSO_3^-$, $CH_3COO^-$, $HCO_3^-$, $H_2PO_4^-$ or $SO_4^{2-}$. However, that found with the solid membrane electrode was $I^-$ >$Br^-$ >$SCN^-$ >$HSO_3^-$ >$Cl^-$ >$ClO_4^-$, $NO_3^-$, $ClO_3^-$, $CH_3COO^-$, $HCO_3^-$, $H_2PO_4^-$ or $SO_4^{2-}$. Again the former was superior.

### Effect of experimental conditions on continuous monitoring

To confirm the results obtained for the above preliminary tests, the experimental variables such as response range, response rate, effect of pH and temperature and interference of other ions were checked in the continuous monitoring system shown in Figure 1. From the results of these studies, it was clear that the results obtained on the batch work correspond to those obtained during continuous monitoring.

Examples of the responses to chloride and nitrate ions for both thiocyanate ion-selective electrodes are shown in Figure 2. With a liquid membrane electrode, less than 2000 ppm chloride ion did not interfere with the determination of $10^{-4}$ M thiocyanate (5.8 ppm as SCN). Relative standard deviation of measurement was less than 6% using a liquid membrane electrode. With a solid membrane electrode however, more than 8 ppm chloride ion was found to interfere with the determination of 5.8 ppm thiocyanate (the error

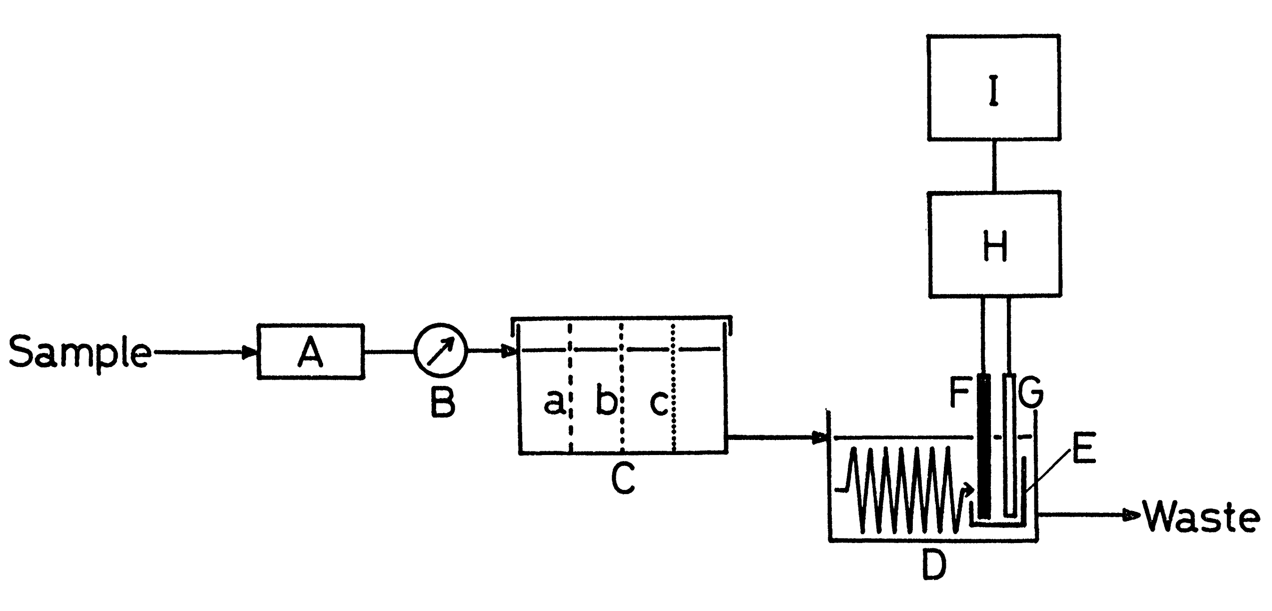

*Figure 1. Schematic diagram of the continuous thiocyanate monitoring equipment.*

obtained was less than 5%, expressed as a relative standard deviation using a solid membrane electrode). It is concluded that the liquid membrane is superior to the solid membrane in this respect.

During continuous monitoring variations in pH had no effect on results. Accordingly, the pH of sample solutions need not be adjusted. Temperature should be accurately maintained at 25±0.5°C to obtain satisfactory results.

**Precision using the continuous monitoring process**

The precision of within-day and day-to-day results obtained with both electrode methods and a spectrophotometric method [3] (see Appendix), is summarised in Table 2. Using $10^{-4}$ M sodium thiocyanate aqueous solution (5.8 ppm as $SCN^-$), day-to-day precision with a liquid membrane electrode demonstrated worse precision than within-day one; day-to-day standard deviation ranged from 1.8 times the corresponding within-day value. Day-to-day precision with a solid membrane electrode demonstrated considerably better precision than that with a liquid membrane electrode as shown in Table 2. Furthermore, day-to-day and within-day precision obtained by the spectrophotometric method demonstrated the highest precision.

However, the author found no difference in precision using both electrodes for continuous monitoring because of poorer overall precision, whereas the spectrophotometric method in this study was considerably more precise on the batch work.

**Continuous monitoring of the waste water samples**

As shown in Figure 1, only the thiocyanate ion-selective electrode with a zephiramine thiocyanate in DCE solution as the liquid membrane solution was applied to the continuous monitoring of thiocyanate in waste water from the Saidaiji Plant of Japan Exlan Co, Ltd. This water sample could not be monitored using a thiocyanate ion-selective electrode with a silver bromide solid membrane since it contained about 2000 ppm chloride ion from sea water.

A typical example of the results for the continuous monitoring of the waste water sample was given in the previous paper [1] where the results obtained by using both the proposed liquid membrane electrode method and the spectrophotometric method [3] were compared in detail to evaluate the reliability of the electrode method as shown in Table 3. The correlation coefficient between the electrode and spectrophotometric methods was found to be significant though the thiocyanate concentrations with the electrode method were a little higher than those with the spectrophotometric method because of a small positive difference based upon the co-existing ions such as chloride and nitrate ions.

In the liquid membrane electrode method described here, thiocyanate present as thiocyanate ion in such water samples could automatically be analysed without any interference from co-existing ions (at 5.8 ppm level of thiocyanate, less than 2000 ppm chloride and less than 6 ppm nitrate did not interfere with this determination as the error incurred was less than 6% which was the relative standard deviation of this method). But for water samples containing less than 8 ppm chloride and more than 6 ppm nitrate, a thiocyanate ion-

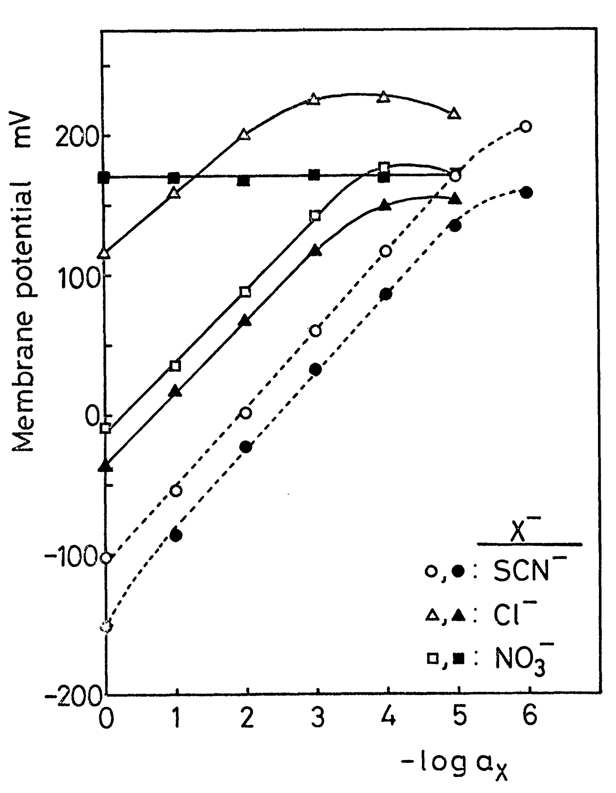

*Figure 2. Electrode response to other ions on continuous monitoring. (○, △, □) Thiocyanate ion-selective electrode with a liquid membrane was used. (●, ▲, ■) Thiocyanate ion-selective electrode with solid membrane (Toa Modal BR-125 bromide ion-selective electrode available commercially) was used.*

**Table 2. Comparison of the precision results for liquid and solid membrane electrode methods and a spectrophotometric method**

| Method | Precision | Control sample | n | mean | sd | rsd(%) |
|---|---|---|---|---|---|---|
| Liquid membrane electrode | Within-day | $10^{-4}$ M Thiocyanate | 16 | 5.8 | $0.2_1$ | 3.6 |
| | | Waste water * | 16 | 18 | 1.0 | 5.6 |
| | Day-to-day | $10^{-4}$ M Thiocyanate | 25 | 5.9 | $0.3_7$ | 6.3 |
| | | Waste water * | 25 | 19 | 1.2 | 6.3 |
| Solid membrane electrode | Within-day | $10^{-4}$ M Thiocyanate | 16 | 5.9 | $0.1_9$ | 3.2 |
| | | Waste water * | – | – | – | – |
| | Day-to-day | $10^{-4}$ M Thiocyanate | 35 | 5.7 | $0.2_6$ | 4.6 |
| | | Waste water * | – | – | – | – |
| Spectrophotometry | Within-day | $10^{-4}$ M Thiocyanate | 8 | 5.8 | $0.0_9$ | 1.6 |
| | | Waste water * | 8 | 17.2 | $0.1_4$ | 0.8 |
| | Day-to-day | $10^{-4}$ M Thiocyanate | 10 | 5.7 | $0.0_1$ | 1.9 |
| | | Waste water * | 10 | 17.5 | $0.3_0$ | 1.7 |

**Waste water sample was obtained from Saidaiji Plant of Japan Exlan Co, Ltd. The sample contained about 2000 ppm chloride and about 6 ppm nitrate, so that the concentration of thiocyanate could not be determined with a thiocyanate ion-selective electrode with solid membrane.*

selective electrode with a silver bromide or thiocyanate as the liquid membrane solution might preferably be used for continuous monitoring of thiocyanate ion.

### Automatic cleaning of the electrode

As shown in Figure 1, an ultrasonic wave cleaner was used to clean the electrode and its operating conditions were studied for the liquid and solid membrane thiocyanate ion-selective electrodes.

It was found that both electrodes were best cleaned by an ultrasonic wave cleaner in order to prevent contamination from suspended solids in waste water samples. When this method was not used, the electrodes had to be washed by a soft brush every day, whereas the electrodes could be used continuously for about three weeks when the ultrasonic wave cleaner was used.

### Life of electrode

To examine day-to-day error, results from the control samples given by a newly constructed electrode on one day were arbitrarily chosen as being correct. Results obtained on other days were related to these expected values. When $10^{-4}$ M sodium thiocyanate aqueous solution (5.8 ppm as $SCN^-$) was used as a control sample, the membrane potential of liquid membrane electrode on day one was 108 mV. The life of the electrode was therefore established by the following criteria. The membrane potential should be within the range 103 to 113 mV and the error compared with the previous day's data should not be more than 6% (relative standard deviation of the liquid membrane electrode method).

It was found that when both electrodes were operating automatically for 24 hr a day in 5.8 ppm thiocyanate control sample solution, the life of a liquid membrane electrode was 15 (variation -3 to +11) days and that of solid membrane electrode was found to be 20 (variation -6 to +10) days without any electrode washings. However, when the actual waste water sample was used, the former rose to 22 (variation -4 to +6) days and the latter to 23 (variation -5 to +4) days even when using an ultrasonic wave cleaner. Even for environmental water samples, contamination caused from suspended solids under the ultrasonic cleaner need not necessarily shorten the lifetime of both types of electrode.

### Maintenance of electrode

In order to check the results, the liquid membrane electrode used in this work should be standardised against a standard sodium thiocyanate aqueous solution ($10^{-4}$ M; 5.8 ppm as $SCN^-$) as a control sample solution every day. Since there was no provision for auto-standardisation, the standardisation was carried out manually each day to guarantee the certainty of the electrode method. If the membrane potential of a liquid membrane electrode used in the standardisation did not range from 103 to 113 mV, the electrode was checked in detail for contamination from suspended solid and so on, even though the samples were filtered with three different meshed plastic filters (first: 10 mesh, second: 40 mesh, third: 200 mesh) and the ultrasonic wave cleaner was used to prevent electrode contamination.

When the electrode did not show a Nernstian relationship and did not recover, the assemblies of the liquid membrane electrode such as the porous membrane filter, the organic liquid membrane and internal reference solutions could be replaced. The solid membrane electrode however has to be exchanged for a new electrode. With the former, in general, the assemblies described above needed renewing at least once a month since the baseline in the recordings was gradually raised. With the latter, the body of the electrode should also be replaced each month as this becomes contaminated by suspended solids in environmental water samples.

### Conclusion

Thiocyanate in environmental water samples could be continuously monitored with satisfactory results according to this procedure using a thiocyanate ion-selective electrode with a liquid membrane of zephiramine thiocyanate in DCE solution. A commercially available bromide ion-selective electrode with a solid membrane of silver bromide was compared with this electrode and both electrodes were then evaluated in detail for continous monitoring of thiocyanate in environmental water samples. The main conclusions reached using the proposed liquid membrane electrode are as follows:

1. The linearity range of electrode response for the liquid membrane electrode is 1 to $10^{-5}$ M thiocyanate (slope = -58 mV). For the solid membrane electrode it is $10^{-1}$ to $10^{-5}$ M thiocyanate (slope = -56 mV).
2. The time needed to achieve constant membrane potentials for $10^{-4}$ M thiocyanate (5.5 ppm as $SCN^-$) is 40 and 80 sec for the liquid and solid membrane electrodes respectively.
3. pH variations have no effect on the membrane potentials over the pH ranges 1 to 13 and 2 to 12 for the liquid and solid membrane electrodes respectively.

**Table 3. Evaluation of the reliability of electrode method**

| Sample * | Date of sampling | | Thiocyanate concentration (as $SCN^-$) ppm | | |
|---|---|---|---|---|---|
| | | | Liquid membrane electrode | Spectrophotometry | Difference |
| 1 | January 20, 1976 | at 10:00 h | 13 | 12.6 | +0.4 |
| 2 | | at 11:00 h | 15 | 14.3 | +0.7 |
| 3 | | at 12:00 h | 18 | 17.0 | +1.0 |
| 4 | | at 13:00 h | 38 | 38 | ±0 |
| 5 | | at 14:00 h | 17 | 15.8 | +1.2 |
| 6 | | at 15:00 h | 16 | 14.7 | +1.3 |
| 7 | | at 16:00 h | 14 | 13.7 | +0.3 |
| 8 | January 21, 1976 | at 10:00 h | 8.8 | 8.1 | +0.7 |
| 9 | | at 12:00 h | 10 | 9.8 | +0.2 |
| 10 | | at 14:00 h | 19 | 16.9 | +2.1 |
| 11 | | at 16:00 h | 12 | 10.5 | +1.5 |
| 12 | January 22, 1976 | at 10:00 h | 11 | 10.0 | +1.0 |
| 13 | | at 12:00 h | 10 | 9.4 | +0.6 |
| 14 | | at 14:00 h | 15 | 14.2 | +0.8 |
| 15 | | at 16:00 h | 15 | 14.1 | +0.9 |
| 16 | January 23, 1976 | at 10:00 h | 7.6 | 7.4 | +0.2 |
| 17 | | at 14:00 h | 9.7 | 9.0 | +0.7 |

** All samples contained less than 2000 ppm chloride, less than 6 ppm nitrate, and no other interfering ions.*

4. The order of selectivity is $ClO_4^- > I^- > SCN^- > NO_3^- > Br^- > ClO_3^- > Cl^- > HSO_3^-$, $CH_3COO^-$, $HCO_3^-$, $H_2PO_4^-$ or $SO_4^{2-}$ for the liquid membrane electrode, and $I^- > Br^- > SCN^- > HSO_3^- > Cl^- > ClO_4^-$, $NO_3^-$, $ClO_3^-$, $CH_3COO^-$, $HCO_3^-$, $H_2PO_4^-$ or $SO_4^{2-}$ for the solid membrane electrode. The life of electrode is $22^{-4}_{+6}$ and $23^{-5}_{+4}$ days for the liquid and solid membrane electrodes respectively, using an ultrasonic wave cleaner. During the continuous monitoring of thiocyanate at 5.8 ppm in actual industrial waste water samples, the maximum permissible concentrations of co-existing chloride and nitrate ions are 2000 ($Cl^-$) and 6 ($NO_3^-$) ppm and 8 ($Cl^-$) and >3000 ($NO_3^-$) ppm for the liquid and solid membrane electrodes respectively.

The effect of temperature, within-day and day-to-day precision, automatic cleaning of electrode, and maintenance of electrode were also evaluated, but the author can find no difference between the liquid and solid membrane electrodes in this respect.

The method of using a thiocyanate ion-selective electrode with liquid membrane recommended here seems to be superior to that of using a cyanide ion-selective electrode for the determination of thiocyanate since the latter has a three stop procedure for a quantitative transformation of thiocyanate into cyanide [4].

It is therefore concluded that for continuous monitoring of thiocyanate in environmental water samples such as natural and waste waters containing less than 2000 ppm chloride and a few ppm nitrate only a liquid membrane electrode gives satisfactory results.

ACKNOWLEDGEMENT

The author is greatly indebted to Professor Kyoji Tôei and Dr Shoji Motomizu of Okayama University for their encouragement, and also to Japan Exlan Co, Ltd for permission to publish this paper.

REFERENCES

[1] Korenaga, T. (1979), *Mikrochimica Acta [Wien]*, II, 455.
[2] Toei, K. and Kawada, K, (1972), *Japan Analyst*, **21**, 1510.
[3] Snell, F.D. and Snell, C.T. (1958), "Colorimetric Methods of Analysis", Vol II, 3rd Edition, Van Nostrand, New York, USA, pp 783.
[4] Nota, G. (1975), *Analytical Chemistry*, **47**, 763.
[5] Ross, J.W. (1976), *Science*, **156**, 1378.

APPENDIX

The chemistry of the spectrophotometric method for the determination of thiocyanate [3] is as follows. A ferric nitrate reagent solution was prepared by dissolving 50 g of the salt in 500 ml of distilled water, adding 25 ml of concentrated nitric acid, and diluting 1 litre with distilled water. To 5 ml of clear filtrate of sample solution, was added 1 ml of the ferric nitrate reagent solution and after 5 min the absorbance of ferric thiocyanate formed at 550 nm was measured using a reagent blank as reference. The concentration of thiocyanate in the sample solution is determined by comparing these results against a calibration curve prepared from a standard sodium thiocyanate solution.

### Notes for Contributors

### Presentation of manuscripts

Manuscripts should be typed (double-spaced) on one side of the paper only and with generous margins. The title should be brief and informative avoiding the word "new" and its synonyms. The full list of authors with their affiliations and full address(es) should appear on the title page. On a separate sheet an abstract of no more than 150 words is required. This should succinctly describe the scope of the contribution and highlight significant findings or innovations. It should be written in a style which can easily be translated into French and German.

*The Concise Oxford Dictionary* and Fowler's *Modern English Usage* (both published by Oxford University Press) should be used as the standard for spelling and grammar. Abbreviations should be limited to those generally recognised, or where a frequently occuring term is abbreviated it should, in the first instance, be explained thus "flow injection analysis (FIA) ..." and the abbreviation used thereafter. Abbreviations, for standard measures and units should follow SI recommendations. There are various publications giving guidance on the use of SI units.

References should be indicated in the text by numerals following the author's name, i.e. Skeggs [6]. On a separate sheet of paper, list all references in numerical order thus:

[6] Skeggs, L.T., *American Journal of Clinical Pathology,* 1959, **28**, 311.

Note that journal titles are given in full. Where there is more than one author, the form Foreman et al. should be used in the text but all authors should be named in the list of references. When reference is made to a chapter in a book the reference should take the following form:

[7] Malmstadt, H.V. in "Topics in Automatic Chemistry" Ed. Stockwell P.B. and Foreman J.K. 1978 Horwood, Chichester, pp. 68-70.

Only work which has been published or has been accepted for publication should be cited. Avoid the citation of documents which are subject to restricted circulation, patent literature, unpublished work and personal communications. The latter can be mentioned in the text in parenthesis.

To illustrate a paper line diagrams are preferred to photographs. Photographs should only be used when they significantly add to the discussion. Diagrams, charts and graphs should be carefully drawn in black ink on stout card or heavy quality tracing paper. Most illustrations are reduced for publication; to allow for this originals should be between 16 and 36 cm wide (the depth must not exceed 50 cm). The lettering of diagrams should be sufficiently clear to withstand reduction. Except in the case of proper names, all lettering should be in lower case print. If photographs are used they must be supplied in the form of clear, unmounted, glossy, black and white prints. "Instant" photographs are not normally acceptable. All illustrations must be identified on the reverse showing the figure number and the author's name.

Each illustration should have a fully explanitory caption. Captions should be typed together on a separate sheet of paper; they must *not* be an inseparable part of the illustration.

